# Glycemia upon admission and mortality in a pediatric intensive care unit

**DOI:** 10.5935/0103-507X.20180068

**Published:** 2018

**Authors:** Luis Miguel Toro-Polo, Ricardo Yannick Ortiz-Lozada, Silvana Lucia Chang-Grozo, Adrian V. Hernandez, Raffo Escalante-Kanashiro, Lely Solari-Zerpa

**Affiliations:** 1 Escuela de Medicina, Universidad Peruana de Ciencias Aplicadas - Lima, Peru.; 2 Sociedad Científica de Estudiantes de Medicina, Universidad Peruana de Ciencias Aplicadas - Lima, Peru.; 3 Instituto Nacional de Salud del Niño - Lima, Peru.; 4 Evidence-Based Practice Center, Hartford Hospital, University of Connecticut -Hartford, United States.

**Keywords:** Hypoglycemia, Hyperglycemia, Infant mortality, Intensive care units, pediatric

## Abstract

**Objectives:**

To analyze the association between glycemia levels upon pediatric intensive care unit admission and mortality in patients hospitalized.

**Methods:**

A retrospective cohort of pediatric intensive care unit patients admitted to the *Instituto Nacional de Salud del Niño* between 2012 and 2013. A Poisson regression model with robust variance was used to quantify the association. Diagnostic test performance evaluation was used to describe the sensitivity, specificity, positive predictive value, negative predictive value and likelihood ratios for each range of glycemia.

**Results:**

In total, 552 patients were included (median age 23 months, age range 5 months to 79.8 months). The mean glycemia level upon admission was 121.3mg/dL (6.73mmol/L). Ninety-two (16.6%) patients died during hospitalization. In multivariable analyses, significant associations were found between glycemia < 65mg/dL (3.61mmol/L) (RR: 2.01, 95%CI 1.14 - 3.53), glycemia > 200mg/dL (> 11.1mmol/L) (RR: 2.91, 95%CI 1.71 - 4.55), malnutrition (RR: 1.53, 95%CI 1.04 - 2.25), mechanical ventilation (RR: 3.71, 95%CI 1.17 - 11.76) and mortality at discharge. There was low sensitivity (between 17.39% and 39.13%) and high specificity (between 49.13% and 91.74%) for different glucose cut-off levels.

**Conclusion:**

There was an increased risk of death at discharge in patients who developed hypoglycemia and hyperglycemia upon admission to the pediatric intensive care unit. Certain glucose ranges (> 200mg/dL (> 11.1mmol/L) and < 65mg/dL (3.61mmol/L)) have high specificity as predictors of death at discharge.

## INTRODUCTION

The mortality in pediatric intensive care units (PICU) is high, and developing countries are the most affected. Campos-Miño et al.^(1)^ found that the average Latin American PICU mortality was 13.29% in contrast to 5% in European countries. In Peru, León et al.^(2)^ in 1996 and Tantaleán et al.^(3)^ studied the mortality at the Instituto Nacional de Salud del Niño (INSN) PICU and found percentages of 26% and 21%, respectively, which also showed marked differences compared with other countries.

The association between hyperglycemia and mortality has been well studied.^(4,5)^ Umpierrez et al.^(6)^ found that hyperglycemia (defined as serum glucose > 126mg/dL (6.99mmol/L)) is common among hospitalized patients and should be considered an important marker of poor clinical response and increased mortality, especially in patients admitted to critical care units.^(5,6)^ In addition, Branco et al. studied the relationship between blood glucose levels and mortality in children with septic shock, finding that a level of glycemia greater than 176mg/dL is associated with a higher risk of death.^(7)^

In contrast, several authors have found that hypoglycemia is the most common alteration in the serum glucose concentration^(8)^ and the most frequent metabolic disorder in childhood.^(9)^ The NICE-SUGAR (Normoglycemia in Intensive Care Evaluation-Survival Using Glucose Algorithm Regulation) study, done in critically ill patients, found an association between moderate and severe hypoglycemia (serum glucose levels of 41 - 70mg/dL (2.28 - 3.89mmol/L) and < 40mg/dL (2.22mmol/L), respectively) and increased risk of death, especially in patients with severe hypoglycemia and in those who sustained hypoglycemia for more than one day.^(10)^

Assessments of the severity, clinical instability and prognosis are the main challenges to be faced in the pediatric intensive care unit, requiring effective and continuous assessment in critically ill patients.^(11)^ Currently, there are several scoring systems that predict mortality in PICUs, such as Pediatric RIsk of Mortality (PRISM), Pediatric Index of Mortality (PIM) and its updates. However, these all have drawbacks, such as a large amount of information being required or a complex mathematical formula to calculate the probability of death,^(12)^ which makes their application complex.^(13)^

It would therefore be useful to have alternative clinical predictors of mortality in the PICUs. Serum glucose is a simple measure, quick and easy to obtain, and therefore meets the criteria for evaluation as a predictor in dynamic environments, such as the PICU.^(14)^ The objective of this study was to determine the PICU admission glycemia levels that are associated with in-hospital mortality.

## METHODS

This study was a retrospective cohort performed at the PICU of the Peruvian INSN between 2012 and 2013. This referral center is a specialized institute of high complexity; its PICU has 23 beds (16 beds for acute and 7 for chronic patients) and recorded 409 hospital discharges in 2012 (34 monthly discharges).^(15)^ The average length of stay (LOS) was 12 days. The Crude Mortality Rate recorded in 2012 in the service was 18.3%, and the Net Mortality Rate, which only considers the subsequent deaths within 48 hours of admission, was 17.8%.^(15)^

The study population consists of children between 29 days and 18 years of age admitted during the mentioned period, and they were categorized by age groups (1 - 6 months, 7 - 12 months, 1 - 5 years, 6 - 15 years old, and > 15 years). Neither patients without serum glucose measurement within the first 24 hours of admission to the PICU nor those who stayed less than that time were considered. Patients without complete information of cause of death or anthropometric data and patients with diagnosis of diabetes mellitus or insulinoma were excluded.

The main outcome was death at discharge of the PICU. The exposure variable was the glucose category, defined as the first serum glucose level measured at admission to the PICU obtained by venipuncture, expressed in mg/dL, taken from the medical registries, and categorized into the following groups:^(16,17)^ Group 1 (< 65mg/dL or 3.61mmol/L), Group 2 (66 - 100mg/dL or 3.66 - 5.55mmol/L), Group 3 (101 - 199mg/dL or 5.61 - 11.04mmol/L), Group 4 (> 200mg/dL or >11.1mmol/L). When more than one glycemia sample was drawn during the first 24 hours, the first one was chosen for the study in all the included patients.

The study was approved by the Ethics Committee of the *Universidad Peruana de Ciencias Aplicadas* and the *Oficina Ejecutiva de Apoyo a la Investigación y Docencia Especializadas* (OEAIDE) from the INSN.

Informed consent was not accomplished as there was no contact with the patients. Medical records were used as a source of information. Data were collected by health workers of the PICU after capacitation. Death of patients was confirmed by clinical history and death certificate. The data were entered into a database in Microsoft Excel 2010, and quality control was performed by double digitization of data.

### Statistical analyses

Data analysis was performed using the statistical package STATA 13.0. A p of < 0.05 was considered significant.

For univariate analyses, the categorical variables were expressed as frequencies (percentages). Continuous variables were described as the means and standard deviations or medians and interquartile ranges.

For bivariate analyses, normality and homogeneity of variances were evaluated using the Shapiro-Wilk test and Levene test, respectively. The comparison of categorical variables was performed using the Chi square test for parametrical variables and Fisher's exact test for nonparametrical variables.

For bivariate and multivariate analyses, Poisson regression models with robust variance were performed. Variables with a p < 0.05 in bivariate analyses were considered for multivariable analyses. Associations were reported as relative risks (RR) and their 95% confidence intervals (CI). Glycemia between 66 - 100mg/dL (3.66 - 5.55mmol/L) (group 2) was considered as the reference group for glucose categories and group etiology respiratory for diagnosis upon admission.

Additionally, the statistical methodology of the diagnostic test was used to indicate the sensitivity, specificity, positive predictive value, negative predictive value and likelihood ratios for each range of glycemia. A 95%CI was also provided.

## RESULTS

**Figure 1:**
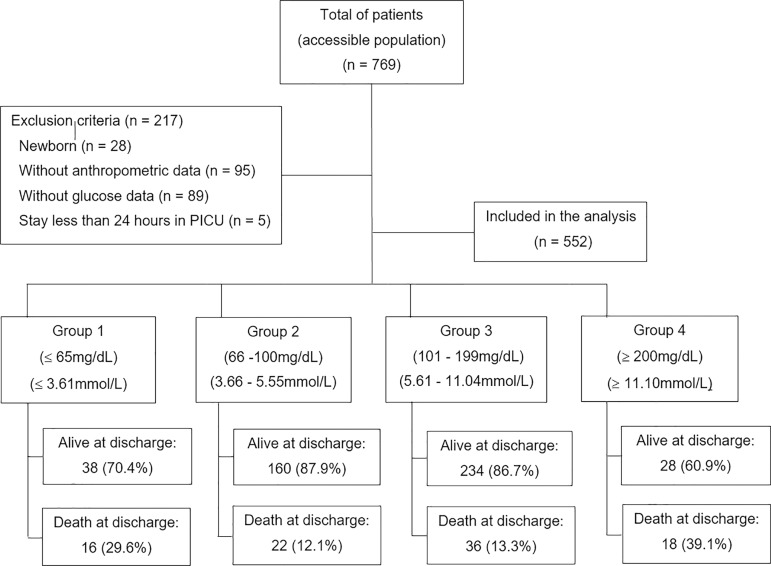
Flow chart of patients included in the analysis. PICU - pediatric intensive care unit.

A census of this population was performed, which was composed of 552 patients. Overall, 769 patients were hospitalized in the PICU during the study period; 217 patients were excluded, including neonates (n = 28), patients who stayed less than 24 hours in the PICU (n = 5), patients lacking anthropometric data (n = 95) and patients lacking glucose data (n = 89), resulting in 552 patients included in the analysis (Figure 1).

The median age was 23 months (IQR = 5 - 79.75), and 52.3% were male. The mean blood glucose level upon admission was 121.30mg/dL (6.73mmol/L). Two hundred seventy (48.9%) patients presented glucose levels in Group 3 (101 - 199mg/dL or 5.61 - 11.04mmol/L), followed by 33.0% in Group 2 (66 - 100mg/dL or 3.66 - 5.55mmol/L). Ninety-two (16.7%) patients died. The most frequent diagnoses upon admission were noncardiovascular surgery (38.5%) and respiratory disease (23.2%). Four hundred sixty-two (83.5%) patients required mechanical ventilation, 14.1% parenteral nutrition and 17.2% presented infection in the PICU (Table 1).

**Table 1:** Demographic and clinical characteristics of the study population (n = 552)

Patient characteristics	Total
Age, months	23 (5.0 - 79.8)
Sex	
Male	289 (52.3)
Glucose	121.30 (70.6)
Glucose group	
< 65 mg/dL	54 (9.78)
(< 3.61 mmol/L)	
66 – 100 mg/dL	182 (33.0)
(3.66 - 5.55 mmol/L)	
101 – 199 mg/dL	270 (48.9)
(5.61 - 11.04 mmol/L)	
> 200 mg/dL	46 (8.3)
(> 11.1 mmol/L)	
Diagnoses upon admission	
Respiratory	128 (23.2)
Infectious	97 (17.6)
Neurological	37 (6.7)
Noncardiovascular surgery	213 (38.6)
Others	77 (14.0)
Mechanical ventilation	462 (83.5)
Obese	4 (0.7)
Malnutrition	114 (20.6)
Eutrophic	434 (78.6)
Infection in the PICU	95 (17.2)
Parenteral nutrition	78 (14.1)
Death at discharge	92 (16.6)

PICU - pediatric intensive care unit. The results expressed as the median (IQR), n (%) or mean (standard deviation).

There was a significant association between glucose groups (p < 0.001), diagnoses upon admission (p < 0.001), nutritional status (p < 0.001), infection in the PICU (p = 0.006) mechanical ventilation (p < 0.001), and death during hospitalization (Table 2).

**Table 2:** Association between patient characteristics and death during hospitalization

Characteristic	Total	Dead	Alive	p value
	n (%)	n (%)	n (%)	
Glucose				< 0.001
< 65mg/dL	54 (9.8)	16 (29.6)	38 (70.4)	
(< 3.61mmol/L)				
66 - 100mg/dL	182 (33.0)	22 (12.1)	160 (87.9)	
(3.66 - 5.55mmol/L)				
101 - 199mg/dL	270 (48.9)	36 (13.3)	234 (86.7)	
(5.61 - 11.04mmol/L)				
> 200mg/dL	46 (8.3)	18 (39.1)	28 (80.9)	
(> 11.1mmol/L)				
Age*				0.330
1 - 6 months	163 (29.5)	35 (21.5)	128 (78.5)	
7 - 12 months	66 (12.0)	12 (18.1)	54 (81.8)	
1 - 5 years	160 (29.0)	21 (13.1)	139 (86.9)	
6 - 15 years	141 (25.5)	21 (14.9)	120 (85.1)	
15 - 18 years	22 (4.0)	3 (13.6)	19 (83.6)	
Sex				0.790
Male	289 (52.4)	47 (16.3)	242 (83.7)	
Female	263 (47.6)	45 (17.1)	218 (82.9)	
Diagnoses upon admission				< 0.001
Respiratory	128 (23.1)	26 (20.3)	102 (79.7)	
Infectious	97 (17.6)	29 (29.9)	68 (70.1)	
Noncardiovascular surgery	213 (38.6)	11 (5.1)	202 (94.8)	
Neurological	37 (6.7)	5 (13.5)	32 (86.4)	
Others	77 (14.0)	21 (27.2)	56 (72.7)	
Nutritional status*				0.009
Obesity	4 (0.7)	0 (0)	4 (100.0)	
Malnutrition	114 (20.6)	33 (29.0)	81 (71.0)	
Eutrophic	434 (78.6)	59 (16.6)	375 (83.4)	
Mechanical ventilation*				< 0.001
Yes	462 (83.5)	89 (19.3)	373 (80.7)	
No	90 (16.5)	3 (3.3)	87 (96.7)	
Parenteral nutrition				0.101
Yes	78 (14.1)	18 (23.1)	60 (76.9)	
No	474 (85.9)	74 (15.6)	400 (84.4)	
Infection in the PICU				0.006
Yes	95 (17.2)	25 (26.3)	70 (73.7)	
No	457 (82.8)	67 (14.7)	390 (85.3)	

PICU - pediatric intensive care unit.

* Fisher’s exact test.

However, multivariate analyses showed that the following variables remained associated with mortality during hospitalization: glucose < 65mg/dL (3.61mmol/L) (RR: 2.01; 95%CI 1.14 - 3.53), glucose > 200mg/dL (> 11.1mmol/L) (RR: 2.91; 95%CI 1.71 - 4.55), malnutrition (RR: 1.53; 95%CI 1.04 - 2.25) and mechanical ventilation (RR: 3.71; 95%CI 1.17 - 11.76) (Table 3).

**Table 3:** Bivariate and multivariate analyses for death during hospitalization

Variable	Crude analyses			Adjusted analyses		
	RR	95%CI	p value	RR	95%CI	p value
Group 1 - < 65mg/dL	2.45	(1.38 - 4.32)	0.002	2.01	(1.14 - 3.53)	0.015
(< 3.61mmol/L)						
Group 2 - 66 – 100mg/ dL	1.00	(Reference)		1.00	(Reference)	
(3.66 - 5.55mmol/L)						
Group 3 - 101 – 199mg /dL	1.10	(0.67 - 1.81)	0.699	1.41	(0.86 - 2.30)	0.172
(5.61 - 11.04 mmol/L)						
Group 4 - >200 mg/dL	3.23	(1.89 - 5.51)	< 0.001	2.91	(1.71 - 4.55)	< 0.001
(> 11.1mmol/L)						
Respiratory diagnosis	1.00	(Reference)		1.00	(Reference)	
Infectious diagnosis	1.47	(0.92 - 2.32)	0.099	1.51	(0.95 - 2.38)	0.076
Noncardiovascular surgery diagnosis	0.25	(0.13 - 0.49)	< 0.001	0.31	(0.15 - 0.63)	0.001
Neurological diagnosis	0.66	(0.27 - 1.61)	0.367	0.85	(0.37 - 1.99)	0.724
Others	1.34	(0.81- 2.21)	0.249	1.22	(0.71 - 2.08)	0.466
Malnutrition	2.14	(1.47 - 3.12)	< 0.001	1.53	(1.04 - 2.25)	0.030
Mechanical ventilation	5.77	(1.86 - 17.85)	0.002	3.71	(1.17 - 11.76)	0.025
Infection in the PICU	1.79	(1.19 - 2.68)	0.004	1.21	(0.79 - 1.86)	0.369

RR – relative risk; 95%CI- 95% confidence interval; PICU - pediatric intensive care unit.

**Figure 2:**
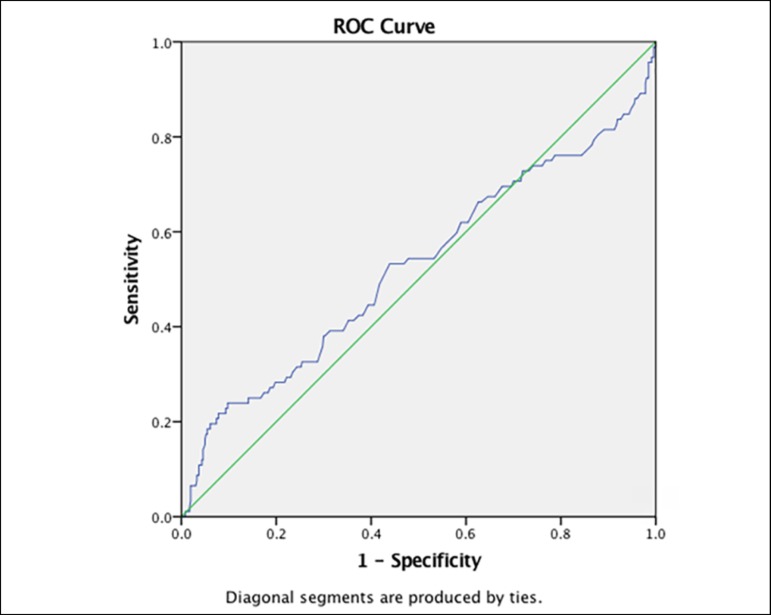
ROC curve.

In the diagnostic test analysis (Table 4), low sensitivity values were found for all ranges of glucose for the prediction of mortality. However, a high specificity (91.7%) was found for glucose values of < 65mg/dL (3.61mmol/L) and for values > 200mg/dL (> 11.1mmol/L) (93.9%). Neither the positive nor the negative likelihood ratios could be considered of significant clinical value. When performing the ROC curve, we found an area under the curve of 0.53 (95%CI 0.45 to 0.60) (Figure 2).

**Table 4:** Performance of the ranges of glucose as predictors of mortality at discharge

Glycemia	Sensibility(%)	Specificity(%)	Positive predictive value	Negative predictive value	Positivelikelihood ratio	Negativelikelihood ratio
≤ 65mg/dL	17.4	91.7	29.6	84.7	2.1	0.9
(< 3,61mmol/L)						
66 - 100mg/dL	23.9	65.2	12.1	81.1	0.7	1.2
(3,66 - 5,55mmol/L)						
101 - 199mg/dL	39.1	49.1	13.3	80.1	0.8	1.2
(5,61 - 11,04mmol/L)						
> 200mg/dL	19.6	93.9	39.1	85.4	3.2	0.9
(> 11,1mmol/L)						

Finally, patients with glucose values < 50mg/dL (2.78mmol/L) had a mortality of 43.4% in comparison with patients with glucose values of ≥ 250mg/dL (13.88mmol/L), who had 37.1% of mortality. As shown, in the extreme ranges of glycemia, hypoglycemia has higher percentages of mortality than hyperglycemia.

## DISCUSSION

Patients with hypoglycemia or hyperglycemia upon admission to the PICU showed an increased risk of death at discharge. The group with the highest risk of death was that with glucose levels > 200mg/dL (> 11.1mmol/L) followed by those with glucose levels < 65mg/dL (3.61mmol/L).

With respect to children with hyperglycemia, these findings are consistent with those of Park et al.,^(16)^ who showed that patients with hyperglycemia > 300mg/dL (> 16.65mmol/L) had a higher death rate at discharge compared to patients who had glycemia between 100 and 199mg/dL (5.61 - 11.04mmol/L). The results are also similar to those made by Klein et al., who studied 1550 hospitalized children in the PICU and concluded that patients with glucose levels > 200mg/dL (> 11.1mmol/L) on the first day of PICU admission had a significantly higher mechanical ventilation time, longer stays in the PICU and lower survival rates compared with those who had normal blood glucose levels.^(18)^

It has been proposed that the liberation of disease-induced stress hormones, such as epinephrine and cortisol, leads to hepatic glycogenolysis mediated by catecholamines, as well as direct sympathetic stimulation of glycogen breakdown that leads to hyperglycemia.^(16)^ Furthermore, use of intravenous dextrose, plus the exogenous use of glucocorticoids and catecholamines, might contribute to an increase in glucose levels. On the other hand, hyperglycemia has multiple effects on the body, such as immunosuppression, which leads to infection, increased blood pressure and natriuretic peptide levels, and platelet hyperactivity, which lead to thrombotic events and neuronal damage that can induce cerebral ischemia and death.^(16)^

The association between hypoglycemia and mortality has been studied previously. A study in the pediatric population at the Befelatanana University Hospital (Madagascar)^(19)^ found that children with hypoglycemia (glycemia < 40mg/dL or 2.22mmol/L) had the highest risk of death (RR: 12.2; 95%CI: 6.2 - 23.7), followed by those with hyperglycemia (glycemia > 150mg/dL or > 8.32mmol/L) (RR: 2.5; 95%CI: 1.0 - 6.2). The authors also found that children with hypoglycemia had a greater decrease in consciousness; increased vomiting; and higher incidence of severe illness, severe dehydration and severe malnutrition. Similarly, Osier et al.^(8)^ in the Kilifi District Hospital in 1999, found that mortality of patients with hypoglycemia was higher than those with normoglycemia, especially in patients with severe signs of disease (prostration or deep breathing) and severe malnutrition. In addition, Egi et al.^(20)^ found similar results regarding the association between hypoglycemia and death in critically ill patients at discharge.

Hypoglycemia causes impairment of autonomic function, release of inflammatory mediators and cytokines, alteration of blood flow and composition, white-cell activation and vasoconstriction. Severe hypoglycemia is associated with a prolonged QT interval and fatal cardiac arrhythmias.^(10)^ These events can answer to a causal relationship, but hypoglycemia can only be a result of disease processes, which are responsible for death yet not the cause.^(10)^ In this case, hypoglycemia could be used as a marker of the predisposition of death.

In contrast to the results found in this study, Blesa Malpica et al.,^(21)^ Freire et al.^(22)^ and Larrondo Muguercia et al.^(23)^ concluded that glycemia during the first twenty-four hours was not a prognostic factor for mortality in critically ill patients. However, they found a linear relationship between elevated levels of glucose and severity of disease. Therefore, they concluded that blood glucose monitoring remains useful and necessary because its dysfunction expresses metabolic instability.

As for other variables associated with mortality in critically ill patients, Sambany et al.^(19)^ found a significant association between hepatomegaly and coma and death. Furthermore, in the study of Srinivasan et al.,^(24)^ infusion of vasoactive substances, such as epinephrine, was found to be associated with mortality. In adults, Freire et al.^(22)^ reported that the severity of illness measured by the Acute Physiology and Chronic Health Evaluation (APACHE) II scale, severe hypoalbuminemia, severe lactic acidosis and mechanical ventilation showed independent associations with mortality.

Regarding the results obtained in the ROC curve, there was a low discrimination capacity. This is due to the objective nature of our study, that is, to find the best hyperglycemia, hyper and hypoglycemia values, which is why a traditional curve is distorted.

One of the strengths of our study is that is serves as a census of the attending pediatric population. In addition, we differentiated between surgical and medical causes in comparison with similar studies. Lastly, to our knowledge, this is the first study in the Latin-American population to address this issue.

Our study had several limitations. Certain variables were not taken into consideration because we were unable to gather their information, which could have influenced the association between glucose and mortality, such as the use of glucocorticoids or exogenous catecholamines, insulin therapy, disease severity measured by usual scales and nutrition 12 hours prior to admission.^(19)^ We also did not consider when certain variables, such as mechanical ventilation, began during the patient's stay in the ICU. Furthermore, an analysis of the excluded patients was not executed.

It would be useful to rely on a marker that can identify patients with increased likelihood of death, therefore achieving better distribution of material and human resources, a goal that becomes more important when referring to developing countries.^(3)^ This marker should be simple, readily available and fast, which would be helpful in a dynamic environment, such as the PICU. Measuring serum glucose meets these characteristics, and thus we evaluated it as a predictor of death at discharge.

## CONCLUSION

Our study showed a significant association between glucose and mortality at both extremes of the spectrum: hyperglycemia and hypoglycemia. Using these ranges of glucose as markers for mortality yielded very high specificities but suboptimal positive likelihood ratios. Pediatric intensivists should perform a careful monitoring of blood glucose, especially during the first twenty-four hours, since alterations in its levels are linked to adverse patient outcomes and to an increased risk of death at discharge.

Likewise, health professionals are recommended to identify the most vulnerable patients, based on the study's findings, to initiate early and effective treatment, thereby preventing and / or reducing mortality.
